# The pathophysiology of traumatic brain injury at a glance

**DOI:** 10.1242/dmm.011585

**Published:** 2013-09-12

**Authors:** Mayumi Prins, Tiffany Greco, Daya Alexander, Christopher C. Giza

**Affiliations:** 1 Department of Neurosurgery, UCLA, Los Angeles, CA 90095, USA; 2 The Interdepartmental Program for Neuroscience, UCLA, CA 92697-3915, USA; 3 The UCLA Brain Injury Research Center, Los Angeles, CA 90095-6901, USA; 4 Department of Neurology, UCLA, Los Angeles, CA 90095, USA

## Abstract

Traumatic brain injury (TBI) is defined as an impact, penetration or rapid movement of the brain within the skull that results in altered mental state. TBI occurs more than any other disease, including breast cancer, AIDS, Parkinson’s disease and multiple sclerosis, and affects all age groups and both genders. In the US and Europe, the magnitude of this epidemic has drawn national attention owing to the publicity received by injured athletes and military personnel. This increased public awareness has uncovered a number of unanswered questions concerning TBI, and we are increasingly aware of the lack of treatment options for a crisis that affects millions. Although each case of TBI is unique and affected individuals display different degrees of injury, different regional patterns of injury and different recovery profiles, this review and accompanying poster aim to illustrate some of the common underlying neurochemical and metabolic responses to TBI. Recognition of these recurrent features could allow elucidation of potential therapeutic targets for early intervention.

## Introduction

Traumatic brain injury (TBI) occurs when a traumatic event causes the brain to move rapidly within the skull, leading to damage. As illustrated in the poster (panel A), the event can be classified as either impact or non-impact, depending on whether the head makes direct contact with an object (impact) or encounters a non-impact force such as blast waves or rapid acceleration and deceleration (non-impact). A TBI occurs every 15 seconds in the US, generating 1.7 million new head injury victims per year. These events are responsible for 50,000 deaths, leave 80,000 individuals with permanent disabilities and cost more than US$77 billion on average per year (Faul et al., 2010). The frequency of brain injury is currently higher than that of any other disease, including complex diseases such as breast cancer, AIDS, Parkinson’s disease and multiple sclerosis. The magnitude of the TBI epidemic is matched only by the sheer complexity of the cerebral pathophysiology involved. Injury severity, type and location, and the individual’s age and gender, all contribute to producing unique brain pathologies, meaning that no two TBIs are the same. Age and injury severity are particularly important in determining outcome. Falls (35%) and motor-vehicle or traffic-related accidents (17%) are the leading causes of moderate to severe TBI in the US (Faul et al., 2010). The incidence of sport-related concussions is estimated to be 130,000 per year among children 5–18 years of age (Cohen et al., 2009). Among active military personnel, blast injury is the most common cause of TBI (Elder and Cristian, 2009).

Currently, the severity of TBI is categorized based on the Glasgow Coma Scale (GCS), in which patients are scored on the basis of clinical symptoms, and the resulting overall score classifies their injury as mild (score: 13–15), moderate (score: 9–12) or severe (score: <9). Symptoms of mild to moderate TBI can include headaches, dizziness, nausea and amnesia; these injuries usually resolve within days to weeks of the insult. However, occasionally these injuries can result in long-term cognitive and behavioral deficits. Furthermore, there is evidence to suggest that moderate to severe TBI, and even repeat mild TBI, might be associated with increased risk of neurodegenerative diseases such as Alzheimer’s disease (Lye and Shores, 2000), chronic traumatic encephalopathy (McKee et al., 2009) and Parkinson’s disease (Hutson et al., 2011).

To study TBI pre-clinically, researchers have developed several experimental animal models to replicate human pathophysiology. TBI models are used to study aspects of primary and secondary brain injury. Primary injury refers to the initial impact that causes the brain to be displaced within the skull. Secondary injuries gradually occur as a consequence of ongoing cellular events that cause further damage. Fluid percussion (FP), controlled cortical impact (CCI) and weight-drop injury are the most commonly used TBI models that can be modulated to generate injuries with characteristics of mild or severe TBI [Table 1 and discussed in more detail in Namjoshi et al. (Namjoshi et al., 2013)]. FP injury is generated by rapid injection of saline through a craniotomy into the epidural space, which causes the brain to move within the skull. CCI injury is produced by the rapid compression of brain tissue by an air-driven piston through a craniotomy. The region of damaged cells within the compressed brain tissue dies, generating a contusion core, which usually develops within days after injury. Finally, the weight-drop model involves the release of a weight from a known height directly onto the closed skull to produce general movement of the brain. All these models can be adjusted to provide various injury severities and the distinct pathophysiology can vary in different models. Despite the overwhelming number of potential permutations for TBI, research has revealed several characteristic physiological responses common to TBI, which are summarized in this review and in the accompanying poster.

**Figure:**
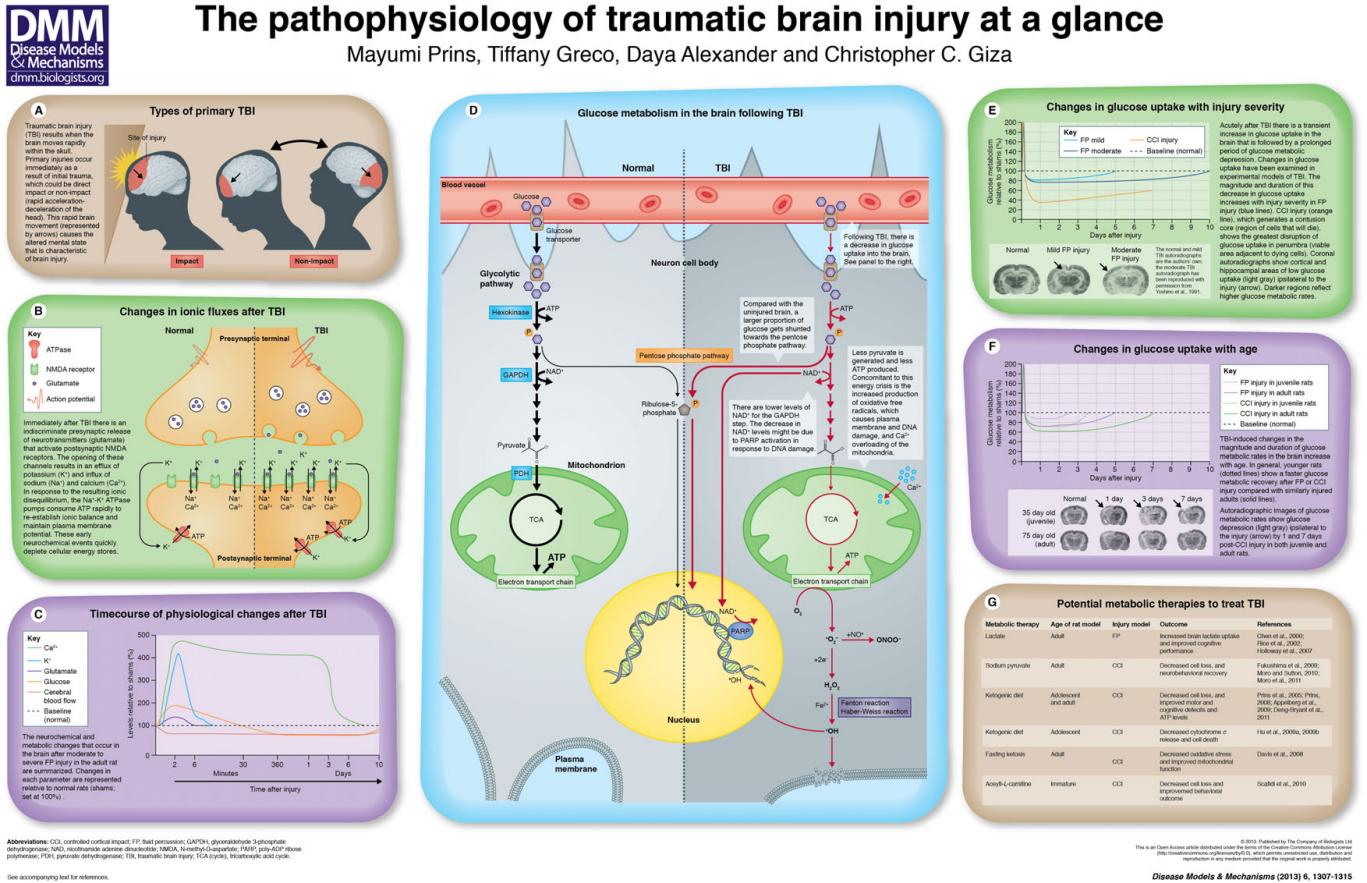


**Table 1.**
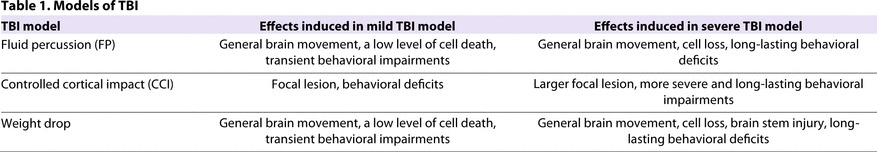
Models of TBI

## Neurochemical changes associated with TBI

Normal transmission of signals involves neurotransmitter-mediated activation of receptors and subsequent controlled ionic changes in the postsynaptic membranes of neurotransmitter-releasing cells. Ionic changes across the bilipid membrane are meticulously regulated by energy-dependent sodium-potassium (Na^+^-K^+^) ATPase pumps, which maintain the membrane potential between −40 and −70 mV (Katsura et al., 1994). TBI induces transient cell membrane disruptions that lead to redistribution of ions and neurotransmitters, altering the membrane potential (see poster, panels B and C). During the acute phase (≤1 hour) after TBI, there is a massive release of glutamate from presynaptic terminals, which disrupts ionic equilibrium on postsynaptic membranes. The amount of potassium (K^+^) released increases with injury severity, as measured by microdialysis (Katayama et al., 1990; Kawamata et al., 1992). In these early studies, mild FP injury produced a 1.4- to 2.2-fold increase in extracellular [K^+^] levels that was blocked by tetrodotoxin (a neurotoxin that prevents brain cell firing), suggesting that this rise in [K^+^] is related to neuronal firing. More severe injuries produced greater increases (4.3- to 5.9-fold) in [K^+^] that were tetrodotoxin-resistant. Administration of kynurenic acid, an antagonist of excitatory amino acids, attenuated the [K^+^] increase in a dose-dependent manner, suggesting that the K^+^ surge is dependent on excitatory neurotransmitters. In order for brain cells to fire again, ionic equilibrium must be re-established, which requires ATP (cellular energy).

In addition to rising [K^+^], calcium (Ca^2+^) accumulation is also commonly observed after TBI (Osteen et al., 2001). Accumulation of intracellular Ca^2+^ activates mitochondrial Ca^2+^ uptake. Ca^2+^ overloading of the mitochondria has been shown to induce oxidative stress and to impair mitochondrial function (Xiong et al., 1997; Peng and Jou, 2010). To determine the effects in the context of TBI, accumulation of isotope-labeled Ca^2+^ (^45^Ca^2+^) was measured in adult rats after FP injury. Increases in ^45^Ca^2+^ were seen as early as 6 hours after the initial injury, and a return to control levels has been observed between 4 days (Fineman et al., 1993) and 7 days (Deshpande et al., 2008) post-injury. Ca^2+^ accumulation corresponded with the presence of cognitive deficits, which were detected using the spatial memory task, the Morris water maze. The surge in Ca^2+^ and the cognitive problems both recovered by 30 days post-injury (Deshpande et al., 2008; Sun et al., 2008). Whereas the influence of animal age on glutamate and K^+^ changes have not been determined after injury, age effects on the pattern of acute ^45^Ca^2+^ accumulation have been observed, with younger animals showing faster normalization after injury and delayed secondary increases associated with cell death compared with older animals (Osteen et al., 2001). In summary, TBI gives rise to early ionic and neurotransmitter perturbations that initiate a cascade of events that disrupt normal cellular function, including changes in glucose metabolism, free radical production and mitochondrial dysfunction.

## Changes in cerebral glucose metabolism in TBI

Glucose is the primary fuel source of the adult brain and its processing through the glycolytic pathway provides carbons for the tricarboxylic acid (TCA) cycle for energy production in the form of ATP. Changes in cerebral glucose metabolism (CMRglc) have been established as a hallmark response after TBI. Two chemical isotopes (^18^F-DG and ^14^C-2DG) are frequently used to measure glucose uptake (transport) into the brain. Glucose analogs are metabolized by the enzyme hexokinase, which is the first enzyme in the glycolytic pathway (see poster, panel D). Once these isotopes are cleaved by hexokinase they become trapped in the cells, which allows the accumulation of glucose to be measured. Autoradiographic images are generated to look at regional changes in isotope-glucose uptake, which can be quantified according to the density of the gray scale. Darker regions reflect greater glucose uptake and whiter regions reflect lower glucose uptake (see poster, panel E). These isotopes have been used to show a rapid increase in glucose uptake followed by a prolonged period of glucose metabolic depression, as observed in both experimental and clinical head injury (Yoshino et al., 1991). Following FP injury in adult rats, the early transient increase in CMRglc can be detected 30 minutes post-injury and it has been shown that this can be attenuated by administration of kynurenic acid (Kawamata et al., 1992). It has been proposed that this initial increase in CMRglc is due to an increased requirement of cellular energy to restore the ionic balance and neuronal membrane potential (Hovda et al., 1990; Hovda et al., 1991; Hovda et al., 1996). This acute period of ‘hyperglycolysis’ has been observed within the first 8 days after severe human head injury (Bergsneider et al., 1997).

The acute period of increased CMRglc is followed by a prolonged period of decreased CMRglc. This decrease in CMRglc has been observed in experimental models of FP and CCI (Andersen and Marmarou, 1992; Yoshino et al., 1991; Kawamata et al., 1992; Richards et al., 2001; Chen et al., 2004), and has also been documented in human TBI (Bergsneider et al., 2000; O’Connell et al., 2005). In the experimental models, the magnitude and duration of this glucose metabolic depression were found to increase with injury severity. Glucose metabolic depression was maintained for 5, 10 or 14 days after mild, moderate or severe FP injury in adult rats, respectively (mild and moderate are shown in panel E of the poster) (Hovda et al., 1994). CCI injury has been found to induce a more profound and longer-lasting depression in the penumbra (area adjacent to the core of cells that will die) than does FP injury (Sutton et al., 1994; Prins and Hovda, 2009). Consistent with the experimental model data, global cortical CMRglc depression was found to be greater in severely injured TBI patients, although this was not closely associated with levels of consciousness (Bergsneider et al., 2000). However, glucose metabolic rates in the thalamus, brain stem and cerebellum significantly correlated positively with the levels of consciousness, as measured by the GCS (Hattori et al., 2003).

Age-related differences in the magnitude and duration of CMRglc depression have been demonstrated in experimental models of TBI (see poster, panel F). Specifically, the duration of glucose metabolic depression increases with age. FP-injured postnatal day (PND) 17 juvenile rats show glucose metabolic recovery to the level of age-matched control animal (sham) levels within 3 days (Thomas et al., 2000), whereas recovery takes approximately 10 days in adult rats. In the diffuse injury model, adult-like patterns in glucose metabolism depression are achieved during adolescence (Prins and Hovda, 2001). Following CCI in the adolescent rat (PND 35), the metabolic rates in subcortical and cortical structures recover within 3 or 5 days, respectively (Prins and Hovda, 2009). A longer-lasting glucose metabolic depression was observed in the cortex among PND 75 (adult) rats with CCI injury, as shown in the poster (panel F). In general, the younger animals seem to show faster reversion of glucose metabolic depression. In humans, there has only been one study that examined glucose uptake after TBI in the pediatric brain (4 months to 19 years of age), but young and adolescent children were not compared with each other or with adults (Worley et al., 1995). Comparison of glucose metabolic changes in TBI between different age groups within the pediatric population, or a comparison between adults and children, has not yet been made in humans. Regardless of age, the prolonged glucose metabolic depression reflects a period of time during which glucose uptake into the brain is compromised. This could cause downstream negative effects if the energy demands of the brain are not sufficiently met.

## Post-TBI energy crisis: causes and consequences

Although decreased glucose uptake after injury seems to be a universal response, the underlying mechanism remains unknown. There are three possibilities: decreased blood availability [which depends on cerebral blood flow (CBF)], defects in glucose transporter function or decreased metabolic demand for glucose. The rapid increase in CMRglc immediately after injury reflects an acute period of increased glucose consumption (as outlined above) and this occurs during a time when CBF has been shown to decline (Golding et al., 1999). In an earlier study, the adult rat brain showed a 52% decrease in cortical CBF within 15 minutes of FB injury, and the level remained significantly low for 4 hours, with recovery occurring at 24 hours post-injury (Yamakami and McIntosh, 1991). Two hours after weight-drop injury, Grundl et al. observed decreases in CBF in both immature and mature animals (Grundl et al., 1994). During this acute phase, when CBF does not meet the cerebral metabolic needs of the tissue, this mismatch or ‘uncoupling’ can initiate cascades of secondary injury events and energy crisis. So, do these CBF changes limit glucose availability? Plasma glucose levels in head injury patients are difficult to determine, because the levels are carefully maintained within specific ranges (Vespa et al., 2006). Experimental studies in both immature and mature rats did not reveal significant changes in plasma glucose supply after TBI and did not suggest substrate limitation (Prins and Hovda, 2009).

Another reason for decreased glucose uptake after TBI could be related to impaired glucose transport through the blood vessels and into brain cells. Adult rat studies have shown decreased neuronal glucose transporter (GLUT1) immunoreactivity 2–4 hours after FP injury (Balabanov et al., 2001); however, the expression of this neuronal glucose transporter is patchy in clinical cases of TBI, i.e. regions with increased and decreased expression are present (Cornford et al., 1996). Hattori et al. examined the ^18^F-DG kinetic changes following moderate to severe TBI in humans and determined that hexokinase activity was globally decreased, with glucose transport impairments occurring specifically within the contusion sites (Hattori et al., 2003). Collectively, the studies indicate that glucose transport across the blood-brain barrier is substantially affected by TBI in both animal and human brains.

Finally, decreases in cerebral glucose levels could reflect problems with glucose metabolism within the cell. There is increasing evidence that the glycolytic processing of glucose is affected by trauma. Following CCI injury, glucose metabolic processing was determined with [1,2 ^13^C]-labeled glucose infusion (Bartnik et al., 2005). Proton nuclear magnetic resonance (NMR) spectroscopy revealed a 9–12% increase in the level of glucose that is shunted towards synthesis of pentoses (used in the synthesis of nucleic acids) via the pentose phosphate pathway at 3.5 hours and 24 hours post-injury (see poster, panel D). Furthermore, nicotinamide dinucleotide (NAD^+^) concentrations have been shown to decrease after injury (Vagnozzi et al., 1999; Satchell et al., 2003), which could be at least partly attributable to increased consumption by a DNA repair enzyme, as described in the subsequent section. NAD^+^ is a necessary cofactor for the glycolytic enzyme GAPDH (glyceraldehyde 3-phosphate dehydrogenase), so reductions in NAD^+^ levels can cause glycolytic inhibition (Sheline et al., 2000). Pyruvate dehydrogenase (PDH) is the enzyme that connects the glycolytic pathway to the mitochondrial TCA cycle. Phosphorylation of the E1 subunit of PDH, which inhibits PDH function and therefore carbon entry into the mitochondria, has been shown to occur at a higher frequency than normal at 24 hours after CCI injury (Xing et al., 2009). These TBI-induced alterations in glycolytic enzyme functioning ultimately decrease the ability of glucose to be efficiently processed for oxidative metabolism, and thereby contribute to the post-TBI energy crisis, reflected by reductions in ATP production (see poster, panel D).

## The role of free radicals in TBI

Increased generation of free radicals is another factor that contributes to the TBI-induced metabolic crisis. Free radicals are molecules with unpaired electrons. These compounds are highly reactive because they attempt to gain electrons from surrounding substances, which can result in cell membrane, protein and DNA damage (see poster, panel D). There are two main families of free radicals: reactive oxygen species (ROS) and reactive nitrogen species (RNS). Production of O_2_^−^ is a consequence of normal metabolism, and this species is a precursor to hydrogen peroxide (H_2_O_2_), which can generate hydroxyl radicals (^•^OH) via the Fenton reaction. The ^•^OH is one of the most reactive chemical species. Reaction of O_2_^−^ with nitric oxide (NO^•^) produces peroxynitrite (ONOO^−^). Normal metabolic activities produce levels of ROS that are well managed by cellular antioxidant defense systems. It is only after cerebral injury that the levels of ROS production overwhelm scavenging systems and result in oxidative damage (O’Connell and Littleton-Kearney, 2013; Kerr et al., 1996; Hall and Braughler, 1993). Mitochondria are a source of free radical generation. During normal metabolism, the TCA cycle generates reducing equivalents to produce ATP. Electrons are normally transferred along the electron transport chain, with only 1–2% of the oxygen generating oxygen radicals at Complex I in the respiratory chain. However, following TBI, changes in the availability of these reducing equivalents is diminished and the production of O_2_^−^ is increased. Concomitantly, TBI-induced intracellular Ca^2+^ accumulation can activate numerous enzymes, including xanthine dehydrogenase, phospholipase A2 and nitric oxide synthase (NOS), which increase O_2_^−^ and NO^•^ production (Lewén et al., 2000).

Despite the short-lived nature of free radicals, their production has been quantified after brain injury. In severely injured mice, hydroxyl radical (^•^OH) production increases 60% within the first minute, peaks at 30 minutes after injury and subsequently decreases (Hall et al., 1993a; Hall et al., 1993b). In contrast with this transient period of ^•^OH formation, severe CCI-injured rats showed a 250% increase in ^•^OH production, which was sustained above baseline for 90 minutes (Marklund et al., 2001). A similar pattern in ^•^OH production has been observed after impact injury in the adult rat (Sen et al., 1993; Sen et al., 1994). Acute increases in NOS have also been reported after FP and CCI injury. FP injury induces NOS expression between 24 and 48 hours in immature vascular smooth muscles and neutrophils (Clark et al., 1996). In adult rats, cortical NOS levels increased between 3 and 7 days, as determined by immunostaining astrocytes and macrophages (Wada et al., 1998). In contrast, CCI injury produced a more rapid increase in nitrate levels, with a peak at 5 minutes and a return to baseline by 6 hours post-injury (Rao et al., 1998).

In addition to direct measurement of radical formation after TBI, indirect evidence can also be quantified via lipid peroxidation, protein nitration and DNA oxidation. Lipid peroxidation occurs when ROS react with polyunsaturated fatty acids, causing conformational changes and membrane permeability (Hall et al., 1993b). Increases in markers of lipid peroxidation have been observed 1–24 hours after weight-drop (Hsiang et al., 1997; Marmarou et al., 1994; Vagnozzi et al., 1999; Lewén and Hillered, 1998; Tyurin et al., 2000) and CCI (Singh et al., 2006) injury. Within the first minute following TBI, lipid peroxidation was shown to increase from an undetectable level to 1.77 nmol/g, and peaked at 2 hours post-TBI (72.3 nmol/g), progressively decreasing thereafter (Lewén and Hillered, 1998). Protein oxidation or nitration products were significantly elevated 30 minutes after CCI injury in adult mice and returned to baseline by 12 hours (Singh et al., 2006). Interestingly, the magnitude and duration of protein oxidative damage was inversely related to injury severity following weight-drop injury in the adult rat (Petronilho et al., 2010). Mild TBI produced a threefold increase in protein carbonyls in the cerebellum, cortex hippocampus and striatum immediately after injury, peaking at 3 hours and remaining elevated at 12 hours. Severe TBI, by contrast, caused decreased levels of protein carbonyls at all time points.

It has also been demonstrated that free radical damage to DNA can occur owing to the activation of DNA repair enzymes that generate lesions. Poly-ADP ribose polymerase (PARP) is a nuclear DNA repair enzyme that consumes NAD^+^ in the presence of single and double DNA strand breaks, and its activity has been shown to increase after various types of brain injury. Pathological activation of PARP has been shown to decrease cytosolic NAD^+^ pools, to inhibit glycolytic processing of glucose and to decrease ATP production (Cipriani et al., 2005; Alano et al., 2010). Evidence of PARP hyperactivation in ischemia and the demonstrated neuroprotective potential of PARP inhibitors encouraged research into the role of PARP in TBI. Satchell et al. reported elevation in PARP1 (a subtype of PARP enzymes) activity 8 hours after CCI injury (Satchell et al., 2003). This activity level remained significantly greater than that in controls for 21 days post-injury. Inhibition of PARP activity decreased the lesion size at 24 hours after FP injury, but did not decrease the number of apoptotic cells (LaPlaca et al., 2001). Partial PARP1 inhibition after CCI injury preserved cellular NAD^+^ concentrations and improved functional performance in the Morris water maze (Satchell et al., 2003).

Although age-dependent effects on free radical production or consequent damage in TBI have not been studied in depth, age-related differences in antioxidant defense systems have been examined. After CCI injury, glutathione peroxidase (GPx) activity was examined in immature (21-day-old) and adult mice (Fan et al., 2003). GPx activity increased at 24 hours in the adult, but no change was observed in the immature brain. This lack of increase in antioxidant response in the younger brain might indicate an increased vulnerability to oxidative damage after injury. Confirmation of this in human TBI patients is currently lacking.

## The central role of mitochondria in TBI

Mitochondria play a crucial role in the pathophysiology and energy crisis associated with brain injury. Accumulation of both Ca^2+^ and ROS after injury, as discussed above, contributes to mitochondrial impairments. Excessive Ca^2+^ influx can overwhelm mitochondrial Ca^2+^-buffering capacity and the rate of efflux by the mitochondrial Na^+^-Ca^2+^ exchanger. The resulting Ca^2+^ accumulation has been shown to contribute to mitochondrial membrane-potential collapse (Vergun et al., 1999). It is clear that Ca^2+^ and oxidative stress act in concert to impair mitochondrial function; however, it is not clear which comes first. One hypothesis is that excessive glutamate stimulation activates NADPH oxidase, thereby generating oxidative stress, which in turn activates PARP1, causing depletion of NAD^+^ stores and ultimately resulting in cell death through inactivation of metabolism (Brennan et al., 2009). An alternative mechanism has been proposed, in which mitochondrial Ca^2+^ accumulation causes membrane-potential compromise, which generates excessive free radicals, leading to activated PARP1, depleted NAD^+^ concentrations and, ultimately, cell death (Duan et al., 2007; Abramov and Duchen, 2008). Although the exact order of events remains controversial, the end result is impaired mitochondrial function, reduced energy production and potential for cell death, which have all been observed following TBI in experimental models. Decreases in coupling of the electron transport chain and oxidative phosphorylation have been shown at 30 minutes after injury, with recovery at 1 hour post-CCI in the adult mouse brain (Singh et al., 2006). More prolonged decreases in respiratory control ratio and Ca^2+^-buffering capacity were also seen between 3 and 72 hours. CCI injuries also resulted in an immediate decrease in ATP concentrations, decreased mitochondrial membrane potential, increased mitochondrial permeability transition and increased ROS production in isolated cortical synaptosomes (Sullivan et al., 1998; Sullivan et al., 1999). Long-lasting changes in mitochondrial respiration have also been reported following CCI injury (Xiong et al., 1997). TBI induced a significant reduction in oxidative phosphorylation at 1 hour post-injury; this decrease persisted for 14 days. In the ipsilateral cortex, greater mitochondrial Ca^2+^ accumulation and lower ATP production were observed. Recurring mitochondrial impairments result in activation of both apoptotic and necrotic pathways, contributing to cell death (Lewén et al., 2001; Robertson, 2004).

Alterations Ca^2+^ in mitochondrial energy metabolism have also been described in the immature rat. TBI resulted in uncoupling of respiration within 1 hour after injury and significant decreases in mitochondrial respiration by 4 hours after injury (Robertson et al., 2007). Several age-associated differences in expression of proteins involved in mitochondrial energy metabolism and cell death pathways exist. For example, the electron transport chain proteins II, III and IV reach adult expression levels within the first few months of life, whereas the proteins I and V reach adult expression levels within 2 weeks of birth (Bates et al., 1994). Respiration-dependent Ca^2+^ uptake is also influenced by age. In mitochondria isolated from immature animals, under normal respiratory conditions, respiration-dependent Ca^2+^ uptake is decreased; however, the opposite occurs under injured conditions (Sharma et al., 2000; Robertson, 2004). Mitochondria from immature rats also show increased susceptibility to apoptosis; this increased susceptibility might be due to increased expression of pro-apoptotic proteins (Soane et al., 2008). These combined differences in protein expression could contribute to increased vulnerability of the younger brain after injury. Depending on the time after injury, these changes in mitochondrial function are potentially reversible if the post-TBI cellular energy crisis can be treated.

## Therapeutic opportunities for TBI

The physiological responses of the brain to TBI are complex and interdependent. Cascades of various neurochemical and metabolic processes affect one another, and, as our understanding grows, so too does our appreciation of this complexity. Collectively, the observed impairments at numerous points in the glycolytic pathway and oxidative metabolism of glucose have prompted researchers to consider what would be the optimal cerebral substrate after head injury (see poster, panel G). Growing evidence has documented the brain’s ability to increase its reliance on alternative substrates under conditions of energy stress [starvation (Dahlquist and Persson, 1976; Hawkins, 1971; Hawkins et al., 1986; Kreis and Ross, 1992), hyperketonemia (Owen et al., 1967; Sokoloff 1973), ischemia (Vannucci and Vannucci, 2000), diabetes (Wahren et al., 1999)]. Indeed, a surge of articles reporting the neuroprotective effects of monocarboxylates (lactate, pyruvate and ketones) has emerged in the last decade. For example, uptake of radioactively labeled lactate within the injury site at 30 minutes after FP injury has been observed, suggesting cellular use of the substrate (Chen et al., 2000). Direct administration of lactate was later shown to improve Morris water maze performance (Rice et al., 2002; Holloway et al., 2007); however, it did not reverse TBI-induced decreases in ATP (Prieto et al., 2011). Administration of three injections of sodium pyruvate spaced 1-hour apart following CCI injury have been shown to attenuate cortical cell loss as measured at 6 hours and 2 weeks post-injury (Fukushima et al., 2009), and this treatment also improves neurobehavioral recovery (Moro and Sutton, 2010; Moro et al., 2011). In addition to these monocarboxylates, ketones have also been shown to confer neuroprotection when provided as an alternative fuel source after TBI. Administering the ketogenic diet after CCI injury reduced cortical contusion volumes, improved motor and cognitive function, and alleviated cellular ATP reductions in the adolescent rat brain (Prins et al., 2005; Prins, 2008; Prins et al., 2010; Appelberg et al., 2009; Deng-Bryant et al., 2011). Ketosis has also been shown to decrease cytochrome *c* release and levels of markers of apoptotic cell death after CCI injury in the juvenile rat (Hu et al., 2009a; Hu et al., 2009b). The fasting of adult animals has also been used to induce ketosis, which has been shown to decrease oxidative stress and Ca^2+^ loading, and to improve mitochondrial oxidative phosphorylation at 24 hours post-CCI injury (Davis et al., 2008).

Addition of ethanol pyruvate to cerebrocortical slices exposed to oxidative stress alleviated ATP decreases and improved cell survival (Zeng et al., 2007). Pyruvate also improved ATP depression, alleviated GAPDH inhibition and prevented cell loss in hippocampal excitotoxicity (Izumi and Zorumski, 2010). Additionally, pyruvate has been shown to have antioxidant properties in M Muller cells (Frenzel et al., 2005) and human neuroblastoma cells (Jagtap et al., 2003). Treatment with acetyl-*L*-carnitine after injury improved behavioral outcomes and decreased lesion volume (Scafidi et al., 2010). Despite the emergence of neuroprotective evidence for alternative substrates, it is important to keep in mind that the multiple foci of physiological disruptions that are generated by TBI require a multi-targeted approach. The monocarboxylates have been demonstrated to improve cellular energetics, decrease production of free radicals and reduce cell death, and the metabolism of these downstream alternative substrates could provide a critical therapeutic approach to TBI (Pan et al., 2012; Prieto et al., 2011; Veech et al., 2001; Veech, 2004). Unfortunately, there have been no clinical trials or application of alternative substrate therapy in TBI. Future studies must address the effectiveness of any therapeutic intervention in various age groups given the known pathophysiological differences in response to TBI.

## Conclusions

TBI is a complex dynamic process that initiates a multitude of cascades of pathological cellular pathways. Its symptomatic presentation varies with each individual, injury type, injury severity, age and gender, making it challenging to diagnose, understand and treat. Research efforts to understand the common underlying neurochemical and metabolic responses to TBI could provide further therapeutic options for early intervention of TBI in patients of all ages.

## Supplementary Material

Supplementary Material
