# Supplementary material for: The pathophysiology of traumatic brain injury at a glance
Source: Dis Model Mech. 2013 Sep 12;6(6):1307–15. doi: 10.1242/dmm.011585 (PMC3820255; doi:10.1242/dmm.011585)
Supplement: Supplementary Material [file supp_6_6_1307__index.html]

The pathophysiology of traumatic brain injury at a glance — Supplementary Material 

# The pathophysiology of traumatic brain injury at a glance

## DMM011585 Supplementary Material

**Files in this Data Supplement:**

- **Supplementary Material PDF**
