# Supplementary material for: The pathophysiology of traumatic brain injury at a glance
Source: Dis Model Mech. 2013 Sep 12;6(6):1307–15. doi: 10.1242/dmm.011585 (PMC3820255; doi:10.1242/dmm.011585)
Supplement: Supplementary Material [file supp_011585_DMM011585.pdf]

The pathophysiology of traumatic brain injury at a glance

Mayumi Prins, Tiffany Greco, Daya Alexander and Christopher C. Giza

A Types of primary TBI

Traumatic brain injury (TBI) results when the brain moves rapidly within the skull. Primary injuries occur immediately as a result of initial trauma, which could be direct impact or non-impact (rapid acceleration-deceleration of the head). This rapid brain movement (represented by arrows) causes the altered mental state that is characteristic of brain injury.

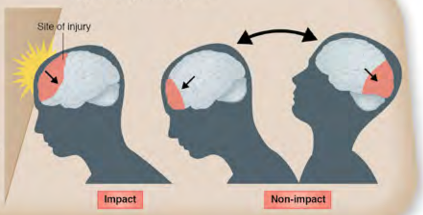

B Changes in ionic fluxes after TBI

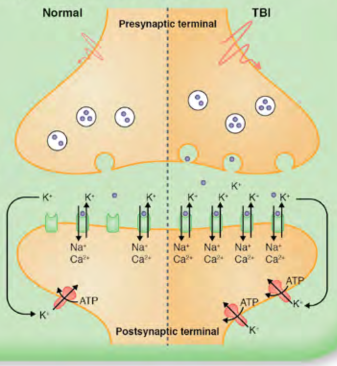

Immediately after TBI there is an indiscriminate presynaptic release of neurotransmitters (glutamate) that activate postsynaptic NMDA receptors. The opening of these channels results in an efflux of potassium (K^+) and influx of sodium (Na^+) and calcium (Ca^{2+}). In response to the resulting ionic disequilibrium, the Na^+-K^+ ATPase pumps consume ATP rapidly to re-establish ionic balance and maintain plasma membrane potential. These early neurochemical events quickly deplete cellular energy stores.

C Timecourse of physiological changes after TBI

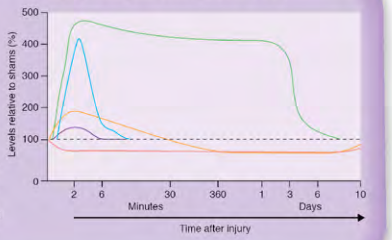

The neurochemical and metabolic changes that occur in the brain after moderate to severe FP injury in the adult rat are summarized. Changes in each parameter are represented relative to normal rats (shams; set at 100%) .

D Glucose metabolism in the brain following TBI

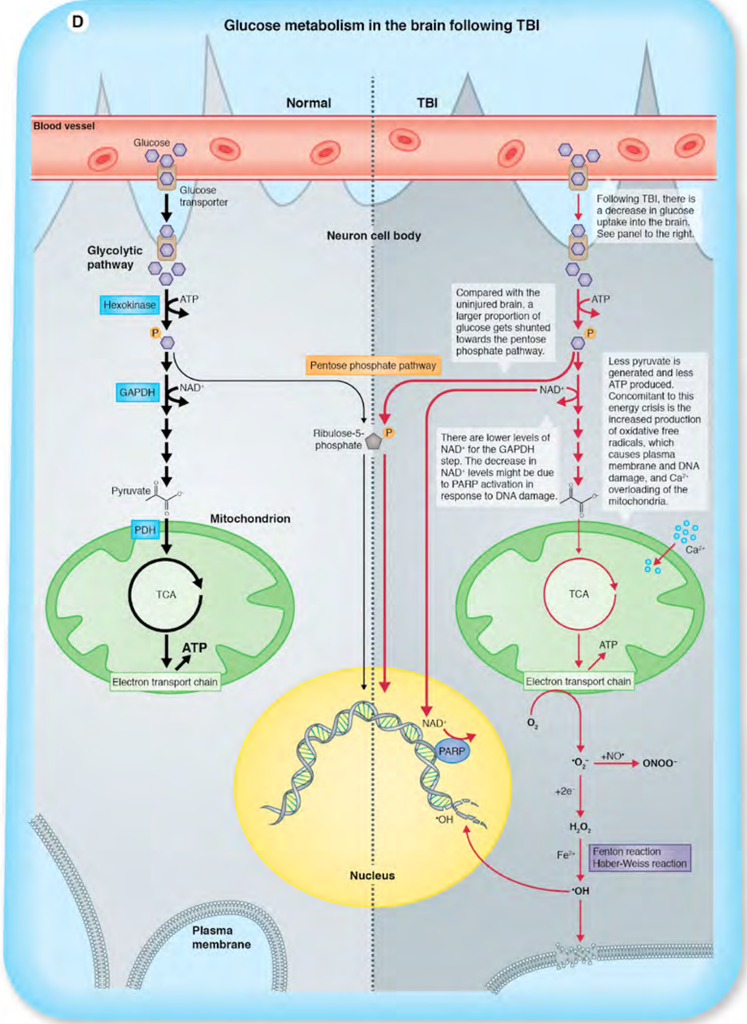

E Changes in glucose uptake with injury severity

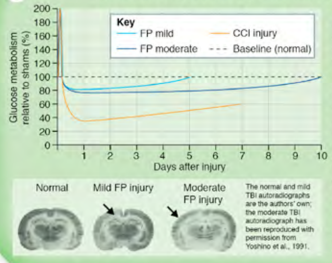

Acutely after TBI there is a transient increase in glucose uptake in the brain that is followed by a prolonged period of glucose metabolic depression. Changes in glucose uptake have been examined in experimental models of TBI. The magnitude and duration of this decrease in glucose uptake increases with injury severity in FP injury (blue lines). CCI injury (orange line), which generates a contusion core (region of cells that will die), shows the greatest disruption of glucose uptake in penumbra (viable area adjacent to dying cells). Coronal autoradiographs show cortical and hippocampal areas of low glucose uptake (light gray) ipsilateral to the injury (arrow). Darker regions reflect higher glucose metabolic rates.

F Changes in glucose uptake with age

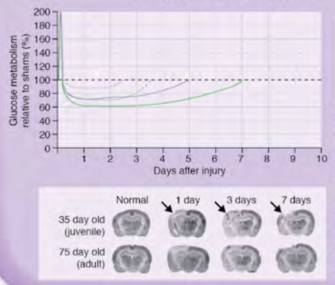

Key
- FP injury in juvenile rats
- FP injury in adult rats
- CCI injury in juvenile rats
- CCI injury in adult rats
- Baseline (normal)

TBI-induced changes in the magnitude and duration of glucose metabolic rates in the brain increase with age. In general, younger rats (dotted lines) show a faster glucose metabolic recovery after FP or CCI injury compared with similarly injured adults (solid lines).

Autoradiographic images of glucose metabolic rates show glucose depression (light gray) ipsilateral to the injury (arrow) by 1 and 7 days post-CCI injury in both juvenile and adult rats.

G Potential metabolic therapies to treat TBI

Metabolic therapy	Age of rat model	Injury model	Outcome	References
Lactate	Adult	FP	Increased brain lactate uptake and improved cognitive performance	Chen et al., 2000; Rice et al., 2002; Holloway et al., 2007
Sodium pyruvate	Adult	CCI	Decreased cell loss, and neurobehavioral recovery	Fukushima et al., 2009; Moro and Sutton, 2010; Moro et al., 2011
Ketogenic diet	Adolescent and adult	CCI	Decreased cell loss, and improved motor and cognitive defecits and ATP levels	Prins et al., 2005; Prins, 2008; Appelberg et al., 2009; Deng-Bryant et al., 2011
Ketogenic diet	Adolescent	CCI	Decreased cytochrome *c* release and cell death	Hu et al., 2009a, 2009b
Fasting ketosis	Adult	CCI	Decreased oxidative stress and improved mitochondrial function	Davis et al., 2008
Acetyl-*L*-carnitine	Immature	CCI	Decreased cell loss and improvemed behavioral outcome	Scafidi et al., 2010

Abbreviations: CCI, controlled cortical impact; FP, fluid percussion; GAPDH, glyceraldehyde 3-phosphate dehydrogenase; NAD, nicotinamide adenine dinucleotide; NMDA, N-methyl-D-aspartate; PARP, poly-ADP ribose polymerase; PDH, pyruvate dehydrogenase; TBI, traumatic brain injury; TCA (cycle), tricarboxylic acid cycle.

See accompanying text for references.

© 2013. Published by The Company of Biologists Ltd
This is an Open Access article distributed under the terms of the Creative Commons Attribution License (http://creativecommons.org/licenses/by/3.0), which permits unrestricted use, distribution and reproduction in any medium provided that the original work is properly attributed.

Disease Models & Mechanisms (2013) 6, doi:10.1242/dmm.011585
